# Proton Therapy Outcomes for Head and Neck Cutaneous Melanoma: Proton Collaborative Group Analysis

**DOI:** 10.14338/IJPT-22-00003.1

**Published:** 2022-07-06

**Authors:** James E. Han, Alicia Lozano, Shaakir Hasan, J. Isabelle Choi, Arpit M. Chhabra, Henry Tsai, Nasiruddin Mohammed, Samir Patel, Sanford Katz, John H. Chang, Charles B. Simone, Robert H. Press

**Affiliations:** 1 Department of Radiation Oncology, New York Proton Center, New York, NY, USA; 2 ProCure Proton Therapy Center, Somerset, NJ, USA; 3 Department of Radiation Oncology, Northwestern University, Chicago, IL, USA; 4 Department of Radiation Oncology, Mayo Clinic, Scottsdale, AZ, USA; 5 Department of Radiation Oncology, Willis Knighton Medical Center, Shreveport, LA, USA; 6 Department of Radiation Oncology, Oklahoma Proton Center, Oklahoma City, OK, USA

## Abstract

**Purpose:**

Reports of proton beam therapy (PBT) utilization for cutaneous melanoma of the head and neck (HN) region is virtually non-existent. This study reports on the efficacy and acute toxicities of PBT for primary HN cutaneous melanoma.

**Materials and Methods:**

We queried the prospectively collected, multi-institutional Proton Collaborative Group registry for all consecutive patients with HN cutaneous melanoma receiving PBT from May 2010 to December 2019. Kaplan-Meier methods were used to estimate overall survival (OS), progression free survival (PFS), and local regional recurrence free survival (LRFS). Toxicity was reported per CTCAE version 4.0.

**Results:**

A total of 8 patients were identified with a median age of 69 (range, 37-88). All patients (100%) underwent surgery followed with postoperative PBT. There were 3 patients (37.5%) with T3 or T4 disease and 4 (50%) with N2 or N3 disease. The median radiation dose was 46 GyRBE (range, 27-70) and median dose per fraction was 2.4 GyRBE (range, 2.0-6.0) with the most common dose fractionation being 44 or 48 GyRBE in 20 fractions (n = 4). At a median follow-up of 40.1 months (range, 1.6-62.4) the 1 and 3 year OS rates were 85.7% and 35.7%, respectively. The median PFS was 25.40 months (95% CI, 2.53-58.70) while PFS at 1 year and 3 years was 85.7% and 35.7%, respectively. LRFS was 100% at 1 year and 85.7% at 3 years. Five of the 8 patients developed distant metastases, of which 3 received immunotherapy. Acute G2+ and G3+ toxicities occurred in 5 of 8 patients and 2 of 8 patients, respectively. G3 toxicities included radiation dermatitis (n = 1) and immunotherapy-related rash (n = 1). No G4+ toxicities were reported.

**Conclusion:**

Single modality PBT for HN melanomas in the definitive setting provides effective and durable local control rates with tolerable acute toxicity. Distant failure remains the primary pattern of failure.

## Introduction

Approximately 1 in 5 new melanoma cases are found in the head and neck (HN) region. Recent analyses have found a 51.1% increase in HN melanoma amongst both adolescents and young adults during the past 2 decades in North America [1]. In addition, the incidence of cutaneous melanomas continues to rapidly increase [2, 3], which has a 5-year overall survival (OS) rate of 80% [4]. The primary treatment of all HN melanomas involves surgical resection with the goal of achieving negative margins [5]. However, when medically inoperable, unresectable, or when surgical specimen findings include high-risk features such as ≥2 lymph nodes (LNs), extracapsular extension, neck LNs ≥4 cm in size, positive surgical margins, and postexcision recurrence, adjuvant radiation therapy (RT) improves local control [6–9]. Despite the decrease in local recurrence with RT, survival remains equivocal due to the high rate of distant metastases.

Proton beam therapy (PBT) is an advanced radiation modality that provides a rapid dose falloff and sharp lateral penumbra that minimizes the integral dose to surrounding organs at risk and increases the therapeutic ratio [10]. These dosimetric improvements have led to the increased use of PBT for HN cancers, and contemporary retrospective series report decreased acute mucositis, nausea, dysgeusia, dysphagia, and narcotic pain requirements [11–13]. However, reports of treatment-related outcomes after PBT for cutaneous melanoma are virtually nonexistent. Therefore, this study sought to evaluate the use and clinical outcomes of PBT for primary HN cutaneous melanoma treated on the multi-institutional prospective Proton Collaborative Group (PCG) database.

## Materials and Methods

### Cohort Definition

The PCG is a clinical research consortium of 18 proton therapy centers in the United States. We queried the prospectively collected, multi-institutional PCG registry for all enrolled patients with primary HN cutaneous melanoma receiving PBT from May 2010 to December 2019. All patients were treated with adjuvant RT or RT with definitive curative intent. Additional patient and treatment information including age, ethnicity, smoking history, Eastern Cooperative Oncology Group (ECOG) score, previous surgery, specifics of PBT, usage of immunotherapy, and TNM staging per the American Joint Committee on Cancer [14] were obtained. Patients were recommended for PBT at the discretion of each individual institutional multi-disciplinary tumor board.

### Study Endpoints

Overall survival, progression-free survival (PFS), and local regional recurrence–free survival (LRFS) were defined as months from end of PBT to the event of interest and censored at the time of last follow-up for patients who remained alive or did not have the event of interest.

Acute toxicity was defined as occurring within 3 months of completing PBT and reported per the Common Terminology Criteria for Adverse Events (CTCAE), version 4.0 (US National Cancer Institute, Bethesda, Maryland). These endpoints were recorded individually by each treating institution and reviewed centrally by PCG staff.

### Statistical Analysis

Baseline patient, tumor, treatment, and outcome characteristics were described in the overall sample—using medians, ranges, counts, and percentages. Survival outcomes (OS, PFS, and LRFS) were estimated in the overall sample and by primary disease groups by using Kaplan-Meier methodology. Descriptive statistics were used to characterize acute toxicities in this cohort. All statistical analyses were performed in SAS version 9.4 (SAS Institute Inc, Cary, North Carolina).

## Results

### Baseline Patient Characteristics, by Treatment Group

Patient characteristics are presented in **Table 1**. A total of 8 patients were identified with a median age of 69 years (range, 37-88 years). All were male (100%) and had ECOG score ≤1 (100%). Fifty percent were white and previous smokers (50%). **Table 2** summarizes treatment and outcome characteristics. All patients (100%) underwent surgery followed with postoperative PBT, of whom 4 had positive surgical margins. There were 3 patients (37.5%) with T3 or 4 disease and 4 (50%) with N2 or 3 disease (**Table 1**). In addition, all 5 patients were treated in the upfront setting and the remaining 3 were treated for subsequent nodal recurrence. Three patients with cutaneous melanoma received immunotherapy for progression of disease.

**Table 1.** Baseline patient and clinical characteristics (N = 8).

**Characteristic**	**Value**
Demographic factors	
Age at diagnosis, median (range), y	69 (37-88)
Sex, No. (%)	
Male	8 (100.0)
Female	0 (0.0)
Ethnicity, No. (%)	
White	4 (50.0)
Other	4 (50.0)
Previous smoker, No. (%)	
Yes	4 (50.0)
No	4 (50.0)
Clinical factors	
ECOG, No. (%)	
0	6 (75.0)
1	2 (25.0)
2	0 (0.0)
Clinical T classification, No. (%)	
T0	1 (12.5)
T1	1 (12.5)
T2	0 (0.0)
T3	1 (12.5)
T4	2 (25.0)
Unknown	3 (37.5)
Clinical N classification, No. (%)	
N0	2 (25.0)
N1	1 (12.5)
N2	2 (25.0)
N3	2 (25.0)
Unknown	1 (12.5)

**Abbreviation:** ECOG, Eastern Cooperative Oncology Group.

Note: Percentages may not sum to 100.0% owing to rounding.

**Table 2.** Treatment and outcome characteristics (N = 8).

**Characteristic**	**Value, No. (%)**
Prior surgery for this cancer	
Yes	8 (100.0)
No	0 (0.0)
Surgery within 4 mo of PBT	
Yes	8 (100.0)
No	0 (0.0)
** **Unknown	0 (0.0)
Margins	
Positive	4 (50.0)
Negative	1 (12.5)
** **Unknown	3 (37.5)
PBT dose (GyRBE), median (range)	46 (27-70)
Follow-up time, median (range), mo	40.1 (1.6-62.4)
Local regional recurrence	
Yes	1 (12.5)
No	7 (87.5)
New progression or metastases	
Yes	5 (62.5)
No	3 (37.5)
Immunotherapy	
Yes	3 (37.5)
No	5 (62.5)
Patient status	
Alive	7 (87.5)
Dead	1 (12.5)

**Abbreviation:** PBT, proton beam therapy.

### Treatment Outcomes

**Figure 1.**
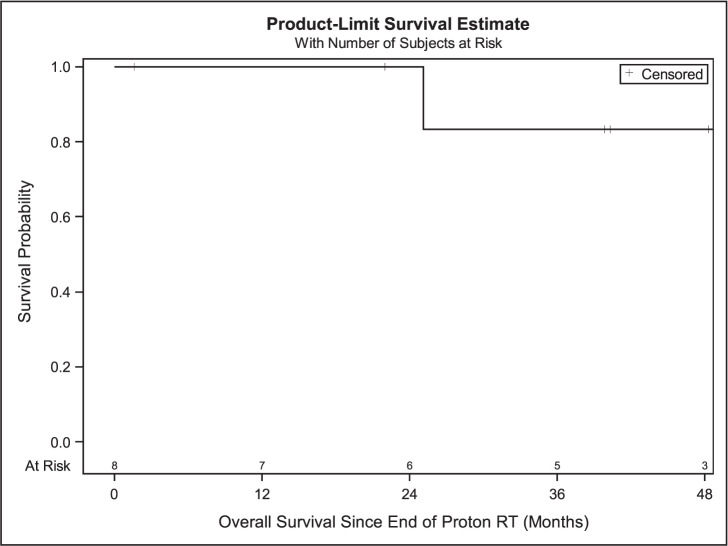
Overall survival in all patients (N = 8). Abbreviation: RT, radiation therapy.

**Figure 2.**
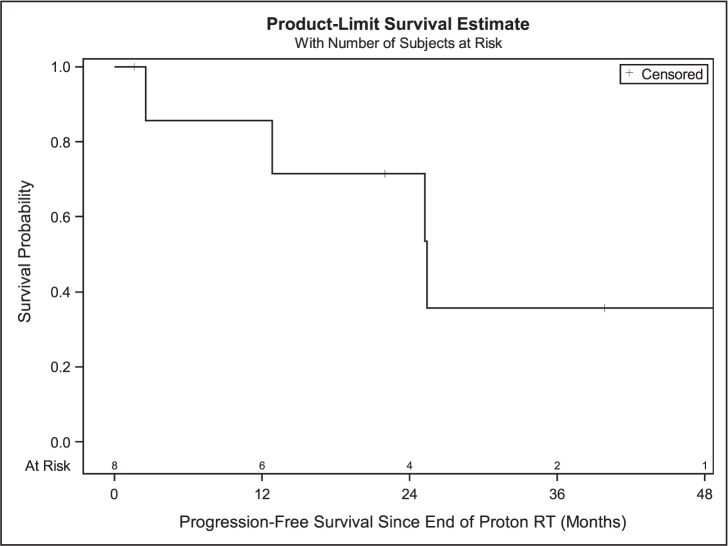
Progression-free survival in all patients (N = 8). Abbreviation: RT, radiation therapy.

**Figure 3.**
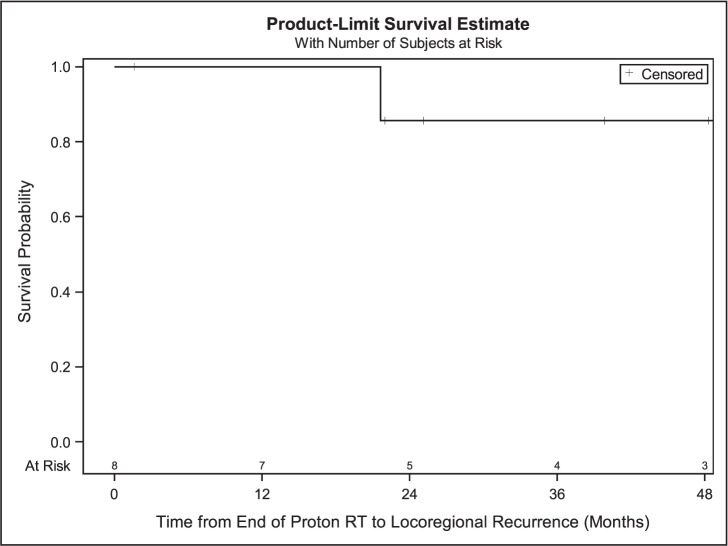
Local regional recurrence among all patients (N = 8). Abbreviation: RT, radiation therapy.

Median follow-up after completion of PBT for all patients was 40.1 months (range, 1.6-62.4) with an estimated median survival of 25.1 months. Median radiation dose was 46 GyRBE (range, 27-70) and median dose per fraction was 2.4 GyRBE (range, 2.0-6.0 ) with the most common dose fractionation being 44 or 48 GyRBE in 20 fractions (N = 4). Other radiation fractionation regimens were 70 GyRBE in 35 fractions (N = 1), 69 GyRBE in 23 fractions (N = 1), 30 GyRBE in 5 fractions (N = 1), and 27 GyRBE in 5 fractions (N = 1). In this cohort, there was 1 death (12.5%), and 5 patients (62.5%) had new progression. One- and 3-year OS rates were 100% and 83.3%. In this cohort, 1 patient with HN cutaneous melanoma (12.5%) had a local regional recurrence (LRR) (**Figure 1**). The median PFS was 25.40 months (95% CI, 2.53-58.70), while PFS at 1 and 3 years was 85.7% and 35.7%, respectively (**Figure 2**). The mean time to LRR was 21.6 months. One-year LRFS was 100% at 1 and 85.7% at 3 years (**Figure 3**). Five of the 8 patients developed distant metastases, of whom 3 received immunotherapy.

### Acute Toxicities

There was a total of 32 acute toxicities among the 8 patients, where 27 (84.4%) were grade (G)1, 3 (9.4%) were G2, and 2 (6.3%) were G3 (**Table 3**). The most common G1 toxicity was radiation dermatitis, which occurred in 4 patients. Four patients had G1 fatigue, while 3 patients had pain and xerostomia (**Table 4**). The G2 toxicities were dysgeusia (n = 1) and radiation dermatitis (n = 2). G3 toxicities included radiation dermatitis (n = 1) and immunotherapy-related rash (n = 1) (**Table 4**). No G4+ toxicities were reported.

**Table 3.** General acute toxicity grades.

**Parameter**	**No. (%)**
Toxicity level data (N = 32)	
Grade 1	27 (84.4)
Grade 2	3 (9.4)
Grade 3	2 (6.3)
Grade 4	0 (0.0)
Patient level data (N = 8)	
Number of acute toxicities, mean (SD)	4.00 (2.14)
Number of acute toxicities, median (range)	3.5 (1-8)

Note: Percentages may not sum to 100.0% owing to rounding.

**Table 4.** Number of specific acute toxicities by grade (N = 32 toxicities among 8 patients).

	**Grade 1**	**Grade 2**	**Grade 3**	**Grade 4**	**Total**
Radiation dermatitis	4	2	1	0	7
Fatigue	4	0	0	0	4
Dysgeusia	2	1	0	0	3
Pain	3	0	0	0	3
Xerostomia	3	0	0	0	3
Dysphagia	1	0	0	0	1
Esophagitis	2	0	0	0	2
Mucositis	1	0	0	0	1
Hoarseness	0	0	0	0	0
Blurry vision	0	0	0	0	0
Cough	1	0	0	0	1
Erythema multiforme	1	0	0	0	1
GERD	0	0	0	0	0
Nasal congestion	1	0	0	0	1
Pharyngitis	0	0	0	0	0
Weight loss	0	0	0	0	0
Anorexia	1	0	0	0	1
Conjunctivitis	0	0	0	0	0
Decreased LOC	0	0	0	0	0
Dyspnea	1	0	0	0	1
Ear and labyrinth disorders	1	0	0	0	1
Nausea	1	0	0	0	1
Papulopustular rash	0	0	1	0	1
Paresthesia	0	0	0	0	0
Postnasal drip	0	0	0	0	0
Respiratory failure	0	0	0	0	0
Watering eyes	0	0	0	0	0
Total	27	3	2	0	32

**Abbreviations:** GERD, Gastroesophageal Reflux Disease; LOC, Loss of Consciousness.

Note: Percentages may not add up to 100 owing to rounding.

## Discussion

In this study, we report outcomes of patients with HN cutaneous melanoma treated with PBT from the prospectively collected multi-institutional PCG registry database. We found that PBT provides durable local control in patients with locally advanced HN melanoma, with most patients treated in the adjuvant setting. The 3-year LRFS, PFS, and OS of patients in our analyses were 85.7%, 35.7%, and 83.3%, respectively, which is comparable if not favorable to reported melanoma literature [6]. Distant failure remains the primary pattern of failure for patients with cutaneous melanoma, and high-grade acute toxicity was rare. To date, this is the largest multi-institutional analyses from North America.

The literature on PBT for HN cutaneous melanoma is sparse. A retrospective series of 47 patients from Mayo Clinic by Bridthikitti et al [15] evaluated the outcomes of the patients with HN skin malignancies including basal cell, melanoma, Merkel cell, angiosarcoma, and others. However, only 3 of these patients had HN melanoma and 2 of these were treated with concurrent and adjuvant nivolumab, while 1 was treated with adjuvant nivolumab following intensity-modulated proton therapy. Dose for definitive and adjuvant RT was 35 Gy in 5 fractions and 30 Gy in 5 fractions, both 2 fractions per week. Two-year estimated LRR and death rate was 11.1% (range, 4.1-30.3) and 12.8%, respectively. However, they did not report outcomes by histology alone. There were no G3+ adverse events during the last week of treatment or at 3-month follow-up appointment.

To the authors' best knowledge, there are no known prior reports of PBT for cutaneous melanoma of the HN region. The lack of prior investigation is likely due to the lack of dosimetric benefit for PBT when treating localized superficial targets. However, treatment of cutaneous melanoma metastatic to the LNs of the parotid gland and/or neck still necessitates comprehensive treatment volumes similar to those used in well-lateralized squamous cell carcinoma or salivary gland cancer. A prior comparative analysis of PBT and intensity-modulated radiation therapy (IMRT) by Romesser et al [11] for unilateral irradiation of cutaneous squamous cell carcinoma or salivary gland cancers suggests PBT results in dosimetric improvement in contralateral organs at risk as well as reductions in acute toxicity. They retrospectively reviewed the cases of 41 patients with major salivary gland cancer or cutaneous squamous cell carcinoma treated with ipsilateral neck radiation with IMRT or PBT. Patients with resectable disease underwent surgery followed by adjuvant RT, while those with inoperable disease received definitive RT. Ipsilateral neck levels Ib-IV were treated in patients with a T3 or 4 primary, positive LNs, high-grade disease, and poorly differentiated histology. Eighteen patients (43.9%) were treated with PBT, and they had significantly lower rates of G2 or greater acute dysgeusia (5.6% vs 65.2%, *P* < .001), mucositis (16.7% vs 52.2%, *P* = .019), and nausea (11.1% vs 56.5%, *P* = .003). However, there was no significant difference in 1-year actuarial local regional control (80.0% vs 95.5%, *P* = .473) or OS (83.3% vs 93.3%, *P* = .083) between PBT and IMRT. One patient treated with PBT developed an in-field parotid bed LRR at 7.7 months, and 1 patient treated with PBT also developed distant metastases. Median follow-up was low at 8.7 months (interquartile range, 4.1-17.6 months).

Despite the small cohort of the current study, the local regional control appears comparable to modern photon melanoma studies. TROG 02.01 [6] was a prospective multi-center randomized trial comparing postoperative RT to observation for high-risk melanoma and reported a lymph-node field relapse rate of 21% at 3 years after photon irradiation. Distant failure remained the primary pattern of failure, similar to our results. Additional comparative studies with larger cohorts and longer follow-up are needed to more clearly define differences in local regional control and late toxicity outcomes between the 2 modalities. However, difficulties exist in the comparison between results, since TROG included multiple disease sites other than the HN region. G2+ toxicity in our analyses compared favorably to the group of HN patients from this trial.

Our results demonstrate adequate local regional control of HN cutaneous melanoma after PBT, suggesting a possible role for PBT when using unilateral nodal treatment volumes for this disease entity, with the rationale of mitigating toxicity in a similar fashion as reported for other HN mucosal carcinomas [11].

The inherent limitations of a retrospective analysis apply to our study despite the prospective nature of the database. The patient population is also small and treatment parameters are heterogeneous, limiting further statistical analysis or extrapolation. Final PBT dosimetry planning was also not available from the PCG database, limiting more detailed assessment of recurrences and/or treatment-related toxicities, and long-term toxicity data were lacking. However, we typically recommend treating in a similar fashion to TROG 02.01, specifically targeting the dissected cervical LN field, lymphadenectomy scar, and including the parotid bed if involved and/or dissected**.**

The advent of immunotherapy and targeted therapies has drastically altered the prognostic landscape for patients with recurrent and metastatic disease. The introduction of ipilimumab in 2011 rapidly altered the systemic treatment of melanoma, leading to improved survival in patients with metastatic disease [16]. In addition, several trials have demonstrated improved recurrence-free survival and distant metastasis–free survival with the addition of adjuvant nivolumab [17] and pembrolizumab [18] in locally advanced resected melanoma. This significant reduction in distant metastases may further underscore the importance of local regional therapy. Most recently, KEYNOTE-716 demonstrated that adjuvant pembrolizumab for the treatment of resected stage III melanoma resulted in improved PFS and OS [19]. At a median follow-up of 20.9 months, the rates of patients with a first recurrence or death were 15% and 24% in the pembrolizumab and placebo groups, respectively. In the context of these advancements in patient outcomes, further prospective investigation into the role of radiation therapy in the immunotherapy era is needed. A phase II trial at MD Anderson Cancer Center (Houston, Texas) is currently enrolling patients to determine the role of RT after sentinel LN-positive melanoma in patients planned for immunotherapy without completion LN dissection.

The literature on the utility of PBT for the treatment of HN cutaneous melanoma is sparse. Furthermore, prospective clinical trials treating HN cutaneous melanoma with PBT is lacking. However, results from a multi-institutional prospectively collected database suggest PBT is safe and effective for cutaneous melanoma of the HN region. Owing to the numerous radiosensitive organs at risk of the HN region, PBT may be an effective tool to mitigate toxicity for select patients, similar to HN cancers with squamous histology. These results in conjunction with prior literature support further investigation of PBT for cutaneous melanoma of the HN. Distant failure remains the primary mode of failure, suggesting more effective systemic therapies are needed for this patient population.

## References

[1] Bray HN, Simpson MC, Zahirsha ZS, Brinkmeier JV, Walen SG, Fosko SW, Osazuwa-Peters N (2019). Head and neck melanoma incidence trends in the pediatric, adolescent, and young adult population of the United States and Canada, 1995-2014. *JAMA Otolaryngol Head Neck Surg*.

[2] McLaughlin CC, Wu X-C, Jemal A, Martin HJ, Roche LM, Chen VW (2005). Incidence of noncutaneous melanomas in the U.S. *Cancer*.

[3] Patrick RJ, Fenske NA, Messina JL (2007). Primary mucosal melanoma. *J Am Acad Dermatol*.

[4] Chang AE, Karnell LH, Menck HR (1998). The national cancer data base report on cutaneous and noncutaneous melanoma: a summary of 84,836 cases from the past decade. *Cancer*.

[5] Rao NG, Yu H-H, Trotti A, Sondak VK (2011). The role of radiation therapy in the management of cutaneous melanoma. *Surg Oncol Clin N Am*.

[6] Henderson MA, Burmeister BH, Ainslie J, Fisher R, di Iulio J, Smithers BM, Hong A, Shannon K, Scolyer RA, Carruthers S, Coventry BJ, Babington S, Duprat J, Hoekstra HJ, Thompson JF (2015). Adjuvant lymph-node field radiotherapy versus observation only in patients with melanoma at high risk of further lymph-node field relapse after lymphadenectomy (ANZMTG 01.02/TROG 02.01): 6-year follow-up of a phase 3, randomised controlled trial. *Lancet Oncol*.

[7] Burmeister BH, Henderson MA, Ainslie J, Fisher R, Di Iulio J, Smithers BM, Hong A, Shannon K, Scolyer RA, Carruthers S, Coventry BJ, Babington S, Duprat J, Hoekstra HJ, Thompson JF (2012). Adjuvant radiotherapy versus observation alone for patients at risk of lymph-node field relapse after therapeutic lymphadenectomy for melanoma: a randomised trial. *Lancet Oncol*.

[8] Ballo MT (2004). Radiotherapy for cutaneous malignant melanoma rationale and indications [discussion in *Oncology (Williston Park)*].

[9] Postow MA, Hamid O, Carvajal RD (2012). Mucosal melanoma: pathogenesis, clinical behavior, and management. *Curr Oncol Rep*.

[10] Mendenhall NP, Malyapa RS, Su Z, Yeung D, Mendenhall WM, Li Z (2011). Proton therapy for head and neck cancer: rationale, potential indications, practical considerations, and current clinical evidence. *Acta Oncol*.

[11] Romesser PB, Cahlon O, Scher E, Zhou Y, Berry SL, Rybkin A, Sine KM, Tang S, Sherman EJ, Wong R, Lee NY (2016). Proton beam radiation therapy results in significantly reduced toxicity compared with intensity-modulated radiation therapy for head and neck tumors that require ipsilateral radiation. *Radiother Oncol*.

[12] van der Laan HP, van de Water TA, van Herpt HE, Christianen MEMC, Bijl HP, Korevaar EW, Rasch CR, van'T Veld AA, van der Schaaf A, Schilstra C, Langendijk JA (2013). The potential of intensity-modulated proton radiotherapy to reduce swallowing dysfunction in the treatment of head and neck cancer: a planning comparative study. *Acta Oncol*.

[13] Manzar GS, Lester SC, Routman DM, Harmsen WS, Petersen MM, Sloan JA, Mundy DW, Hunzeker AE, Amundson AC, Anderson JL, Patel SH, Garces YI, Halyard MY, McGee LA, Neben-Wittich MA, Ma DJ, Frank SJ, Whitaker TJ, Foote RL (2020). Comparative analysis of acute toxicities and patient reported outcomes between intensity-modulated proton therapy (IMPT) and volumetric modulated arc therapy (VMAT) for the treatment of oropharyngeal cancer. *Radiother Oncol*.

[14] Amin MB, Edge S, Greene F, Byrd DR, Brookland RK, Washington MK, Gershenwald JE, Compton CC, Hess KR, Sullivan DC, Jessup JM, Brierley JD, Gaspar LE, Schilsky RL, Balch CM, Winchester DP, Asare EA, Madera M, Gress DM, Meyer LR (2017). American Joint Committee on Cancer *AJCC Cancer Staging Manual* 8th ed.

[15] Bridthikitti J, Viehman J, Harmsen WS, Amundson A, Shiraishi S, Mundy D, Rwigema JCM, Mcgee LA, Patel SH, Routman DM, Lester SC, Neben-Wittich MA, Garces Y, Ma DJ, Foote RL, Birgi SD, Akyurek S, Arslan Y, Birgi E, Bakirarar B, Gumustepe E, Gokce SC (2020). Oncologic outcomes for skin malignancies of the head and neck treated with protons, photons or electrons. *Int J Radiat Oncol Biol Phys*.

[16] Hodi FS, O'Day SJ, McDermott DF, Weber RW, Sosman JA, Haanen JB, Gonzalez R, Robert C, Schadendorf D, Hassel JC, Akerley W, van den Eertwegh AJM, Lutzky J, Lorigan P, Vaubel JM, Linette GP, Hogg D, Ottensmeier CH, Lebbé C, Peschel C, Quirt I, Clark JI, Wolchok JD, Weber JS, Tian J, Yellin MJ, Nichol GM, Hoos A, Urba WJ (2010). Improved survival with ipilimumab in patients with metastatic melanoma. *N Engl J Med*.

[17] Weber J, Mandala M, del Vecchio M, Gogas HJ, Arance AM, Cowey CL, Dalle S, Schenker M, Chiarion-Sileni V, Marquez-Rodas I, Grob J-J, Butler MO, Middleton MR, Maio M, Atkinson V, Queirolo P, Gonzalez R, Kudchadkar RR, Smylie M, Meyer N, Mortier L, Atkins MB, v Long G, Bhatia S, Lebbé C, Rutkowski P, Yokota K, Yamazaki N, Kim TM, de Pril V, Sabater J, Qureshi A, Larkin J, Ascierto PA (2017). Adjuvant nivolumab versus npilimumab in resected stage III or IV melanoma. *N Engl J Med*.

[18] Eggermont AMM, Blank CU, Mandala M, v Long G, Atkinson V, Dalle S, Haydon A, Lichinitser M, Khattak A, Carlino MS, Sandhu S, Larkin J, Puig S, Ascierto PA, Rutkowski P, Schadendorf D, Koornstra R, Hernandez-Aya L, Maio M, van den Eertwegh AJM, Grob J-J, Gutzmer R, Jamal R, Lorigan P, Ibrahim N, Marreaud S, van Akkooi ACJ, Suciu S, Robert C (2018). Adjuvant pembrolizumab versus placebo in resected stage III melanoma. *N Engl J Med*.

[19] Luke JJ, Rutkowski P, Queirolo P, del Vecchio M, Mackiewicz J, Chiarion-Sileni V, de la Cruz Merino L, Khattak MA, Schadendorf D, v Long G, Ascierto PA, Mandala M, de Galitiis F, Haydon A, Dummer R, Grob J-J, Carlino MS, Mohr P, Poklepovic A, Sondak VK, Scolyer RA, Kirkwood JM, Chen K, Diede SJ, Ahsan S, Ibrahim N, Eggermont AMM (2022). Pembrolizumab versus placebo as adjuvant therapy in completely resected stage IIB or IIC melanoma (KEYNOTE-716): a randomised, double-blind, phase 3 trial. *Lancet*.

